# Development of Novel Topical Cosmeceutical Formulations from *Nigella sativa* L. with Antimicrobial Activity against Acne-Causing Microorganisms

**DOI:** 10.1155/2019/5985207

**Published:** 2019-08-14

**Authors:** Nirmani Wishwakala Nawarathne, Kanchana Wijesekera, Weerasinghe Mudiyanselage Dilip Gaya Bandara Wijayaratne, Mayuri Napagoda

**Affiliations:** ^1^Department of Pharmacy, Faculty of Allied Health Sciences, University of Ruhuna, Galle 80000, Sri Lanka; ^2^Department of Microbiology, Faculty of Medicine, University of Ruhuna, Galle 80000, Sri Lanka; ^3^Department of Biochemistry, Faculty of Medicine, University of Ruhuna, Galle 80000, Sri Lanka

## Abstract

Acne vulgaris occurs due to the inflammation of sebaceous follicles in the skin. It is triggered by the activity of some bacterial species like *Propionibacterium acnes*, *Staphylococcus aureus*, and *Staphylococcus epidermidis*. Acquisition of antibiotic resistance by these microorganisms and adverse effects associated with the current treatment regimens necessitate the introduction of novel therapeutic agents for acne vulgaris. Thus, this study was undertaken to develop novel gel formulations from seeds of *Nigella sativa* L. and to evaluate the antibacterial potential against some acne-causing bacterial species. The antibacterial activity of seed extracts was initially screened against *S. aureus* and *P. acnes* by the agar well diffusion method. Thereafter, topical gels were formulated incorporating the ethyl acetate extract of seeds of *N. sativa* at three different concentrations. These topical formulations were subjected to antimicrobial activity studies while the stability was evaluated over a period of 30 days. All three formulations were capable of inhibiting the growth of *S. aureus* and *P. acnes*, with the highest antibacterial activity in the formulation comprising 15% of the seed extract. Interestingly, the antibacterial potency of this formulation against *S. aureus* surpassed the commercial synthetic product used as the positive control. Moreover, any alteration in color, odor, homogeneity, washability, consistency, and pH was not observed while the antibacterial potency was also retained during the storage period. The potent antibacterial activity in topical gel formulations developed from the ethyl acetate extract of *N. sativa* signposts their suitability as alternatives to existing antiacne agents in the management of acne vulgaris.

## 1. Introduction

Acne vulgaris is a dermatological condition that affects over 80% of teenagers and over 10% of adults irrespective of gender. It results because of chronic inflammation of a sebaceous follicle and is characterized by the formation of seborrhea, inflammatory lesions, comedones, and nodules [1, 2]. The recent investigations suggest a possible pathogenic role for the bacterial species, *Propionibacterium acnes*, *Staphylococcus epidermidis*, and *Staphylococcus aureus* in acne vulgaris. Especially, *P. acnes* which is an obligate anaerobic microorganism is responsible for the development of inflammatory acne due to its ability to activate complements and metabolize sebaceous triglycerides into fatty acids, which then chemotactically attract neutrophils. On the other hand, the aerobic *Staphylococcus* species is usually involved in superficial infections within the sebaceous unit in the skin [3].

Current treatment approaches for acne vulgaris include either topical application of benzoyl peroxide, retinoids, and antibiotics such as erythromycin or clindamycin or use of oral medications like retinoids and antibiotics of tetracycline and macrolides classes. However, in the case of severe acne, combinational treatments are normally employed. Although antibiotics are capable of inhibiting inflammation indicative of acne as well as target *P. acnes*, the acquisition of antibiotic resistance by *P. acnes* and other acne-causing bacterial species demands the development of novel therapeutic agents [4]. In this respect, natural ingredients which have been used in traditional systems of medicine such as different parts of plants, spices and condiments, and minerals could be investigated as potent sources for novel antiacne agents.

Apart from their role as flavor enhancers and coloring agents, spices are revered for different pharmacological properties like analgesic, antipyretic, anti-inflammatory, antimicrobial, and anticancer effects [5], and particularly, spices such as anise, mustard, saffron, and cinnamon are reported for their potential applications in pharmacology and cosmetology [6]. Among the spices, *Nigella sativa* L. (black cumin) is of immense importance due to its diverse applications in dermatology and in other pathological conditions [7]. For example, various bioactivities such as antimicrobial [8], immunomodulatory [9], antioxidant [10], anti-inflammatory [11], and antitumor activity [12] have been reported in *N. sativa* seeds and oil, and the bioactivities were mainly attributed to the presence of thymoquinone [13]. The seeds of *N. sativa* have been used for centuries by different cultures in the treatment of several dermatological disorders like acne vulgaris, burns, wounds, and other skin inflammatory conditions [7]. These traditional claims were supported with a clinical study that revealed 20% of *N. sativa* oil extract in lotion formulation had a better efficacy and was less harmful than benzoyl peroxide lotion 5%, which is the basic treatment for mild to moderate stage of acne vulgaris [14]. Furthermore, *N. sativa* is widely utilized in folklore medicine in Sri Lanka as a dermatological remedy and is an important constituent in several topical formulations employed in traditional medicine to treat acne vulgaris [15], suggesting possible antiacne effects. However, scientific investigations are scarce in Sri Lanka to support these claims of indigenous medical practitioners and thereby to develop herbal antiacne formulations in commercial scale. Therefore, the present investigation was undertaken to develop topical cosmeceutical formulations incorporating *N. sativa* and, thereafter, to evaluate the antibacterial activity of those formulations against selected acne-causing bacteria. Thus, we believe that the present study would rationalize the traditional utility of *N. sativa* as an antiacne agent while providing valuable information towards the development of alternative antiacne agents in commercial scale.

## 2. Methods

### 2.1. Plant Material

The seeds of *N. sativa* were purchased and authenticated from a government registered Ayurvedic chemical laboratory (registered number-Ayur/Nish/Put/09) in Chilaw, North Western Province, Sri Lanka. A voucher specimen (MN_2018_003) was deposited at the Department of Biochemistry, Faculty of Medicine, University of Ruhuna, Sri Lanka. The dried seeds (20 g) were initially soaked in hexane, ethyl acetate, and methanol (300–400 mL) separately for 24 hours in a shaker, and thereafter, the solvents were evaporated using a rotary evaporator (HS-2005V-N, South Korea). These crude extracts were subjected to antibacterial activity studies against *S. aureus* in order to select the best solvent for large-scale extraction.

### 2.2. Preliminary Screening of Crude Extracts Prepared from Seeds of *N. sativa* for Antibacterial Activity against *S*. *aureus*

The agar well diffusion method was used to determine the antibacterial activity against *S. aureus* following the method described by Soyza et al. [16] with slight modifications.

Mueller Hinton Agar (MHA) plates were inoculated with a saline suspension of bacteria prepared using isolated colonies of one-day-old pure cultures of *S. aureus* (ATCC 25923) obtained from the Department of Microbiology, Faculty of Medicine, University of Ruhuna, Sri Lanka. The turbidity of the bacterial suspension was adjusted to that of McFarland 0.5 standard. Thereafter, wells (6 mm diameter and 5 mm depth) were prepared in these culture plates at equidistance using a sterilized cork borer. The wells were filled with 50 *μ*L of each of the test solutions (20 mg/mL of hexane, dichloromethane, and ethyl acetate extracts dissolved in 2% DMSO) separately. Thereafter, the plates were incubated at 37°C overnight, and the zone of inhibition around each well was measured. Co-amoxiclav was used as the positive control while 2% DMSO was used as the negative control. The zones >6 mm were considered as inhibitions resulting from significant antibacterial activity. The experiments were performed in duplicate, and the diameter of the zone of inhibition was expressed as mean ± SD.

Depending on the diameter of the zone of inhibition, the best solvent for the large-scale extraction was selected. Further, the minimum inhibitory concentration (MIC) of the above selected extract was determined by the broth microdilution method in 96-well microtitre plates following the method described by Napagoda et al. [17] with slight modifications where resazurin was used for the visual identification of the lowest concentration of test agent that prevents the growth of a bacterium. The MIC values were confirmed by subculturing the content of the above microtitre plate wells in agar plates which also gave an indication of the MBC (minimum bactericidal concentration) values. The assay was conducted in triplicate.

### 2.3. Large-Scale Extraction of *N. sativa*

Considering the results of the preliminary antibacterial screening against *S. aureus*, ethyl acetate was selected as the solvent of choice for the large-scale extraction of seeds of *N. sativa*. Here, 500 g of dried seed materials was extracted with ethyl acetate (2.5 L) for 24 hours in a shaker, and thereafter, the solvents were evaporated using a rotary evaporator (HS-2005V-N, South Korea). This extract was used to prepare the topical gel formulations.

### 2.4. Qualitative Screening for the Phytochemical Constituents in the Ethyl Acetate Extract of Seeds of *N. sativa*

The following standard qualitative screening tests [18] were performed for the identification of different classes of phytochemicals in the ethyl acetate extract of seeds of *N. sativa.* All the tests were conducted along with a suitable positive control.

#### 2.4.1. Test for Alkaloids

Plant extract was dissolved in hydrochloric acid, and then few drops of Mayer's reagent were added and observed for the color change. Creamish precipitate indicates the presence of alkaloids.

#### 2.4.2. Test for Phenolic Compounds

Ferric chloride solution (3-4 drops) was added to the plant extract and observed for the appearance of bluish-black color, which is an indication for the presence of phenolic compounds.

#### 2.4.3. Test for Flavonoids

A small quantity of the extract was heated with ethyl acetate (10 mL) in boiling water for 3 minutes. The mixture was filtered, and the filtrate was shaken with 1 mL of dilute ammonia solution (1%). The layers were allowed to separate and observed for the color change. Yellow coloration in the ammonia layer indicates the presence of flavonoids.

#### 2.4.4. Test for Saponins

Distilled water (6 mL) was added to the extract (2 mL) and shaken vigorously. Formation of persistent foam indicates the presence of saponins.

#### 2.4.5. Test for Terpenoids

Plant extract was taken to a test tube and chloroform was added, and then the test tube was held at 45°. Thereafter, concentrated sulfuric acid was added using a dropper along the wall of the test tube and observed for color change. A reddish-brown coloration of the interface is an indication for the presence of terpenoids.

### 2.5. Preparation of Topical Gel Formulation

Three formulations were prepared by incorporating the ethyl acetate extract of seeds of *N. sativa* at different concentrations, as indicated in Table 1. These concentrations were selected based on the amount of *N. sativa* used in traditional herbal preparations. In order to ensure that the incorporation of above concentrations of ethyl acetate extract for the gel formulations has an advantage of inhibiting the bacterial growth, the antibacterial activity of the extract at these concentrations was evaluated by the agar well diffusion method against *S. aureus* (as mentioned in Section 2.2) and *P. acnes* (under anaerobic conditions, against erythromycin as the positive control).

The antiacne gel base was formulated by using carbopol 940, glycerin, phenoxyethanol, EDTA, rosewater, cetyl alcohol, fuller's earth, polyethylene glycol, and triethanolamine. Carbopol 940 was dissolved in rosewater until it gets completely soaked. Glycerin, phenoxyethanol, EDTA, rosewater, cetyl alcohol, fuller's earth, polyethylene glycol, and triethanolamine were added to the carbopol mixture while stirring in a vortex. Thereafter, the seed extract was incorporated into this mixture at varying percentages.

### 2.6. Antibacterial Activity Studies for the Gel Formulations

All the gel formulations were dissolved in methanol for the determination of the antibacterial activity against *S. aureus* and *P. acnes*.

The agar well diffusion method was employed to determine the antibacterial effect of the gel formulations against *S. aureus* following the method described in Section 2.2. A synthetic commercial antiacne gel was used as the positive control, and the gel base and methanol were used as the negative controls. Thereafter, the MIC of these formulations was determined by the broth microdilution method in 96-well microtitre plates following the method described in Section 2.2. The assay was conducted in triplicate.

The agar well diffusion assay was employed under anaerobic conditions for the determination of antibacterial activity against *P. acnes*. Wells (6 mm diameter and 5 mm depth) were prepared using a sterilized cork borer in the blood agar plates which have been inoculated with clinical isolates of *P. acnes* obtained from the Medical Research Institute, Sri Lanka. The wells were filled with each of the test formulations, and the agar plates were incubated at 37°C for 48 hours in an anaerobic jar and the zones of inhibition were measured after the incubation. A commercial antiacne gel was used as the positive control while the gel base and methanol were used as the negative controls. The experiment was conducted in triplicate.

### 2.7. Stability of the Physical Parameters and Antibacterial Effect of the Formulations

The stability of the physical parameters (color, odor, homogeneity, washability, consistency, and pH) of all three formulations was evaluated at day 30 after the formulation of gels (storage conditions: temperature 30 ± 2°C and relative humidity 75 ± 5%). The antibacterial activity against *S. aureus* was also evaluated at day 30 in order to determine whether the gel formulations are capable of retaining their antibacterial potential over a period of time during storage.

### 2.8. Acute Local Irritation Test with the Optimized Gel Formulation

Acute local irritation test was carried out for 50 human volunteers in the age group of 18–30 years with acne vulgaris. The optimized gel formulation was applied on the right ear lobe of each individual, and signs of hypersensitivity reactions (pruritus, oedema, and erythema) were observed after 30 min.

The ethical approval was obtained from the Ethical Review Committee of Faculty of Medicine, University of Ruhuna, Sri Lanka.

## 3. Results

### 3.1. Preliminary Screening of Crude Extracts for Antibacterial Activity

Among the three crude extracts tested, the highest activity against *S. aureus* was observed in the ethyl acetate extract with a zone of inhibition of 12 ± 0.0 mm in diameter. The zones of inhibition of 7 ± 0.0 mm in diameter were detected for both hexane and methanol extracts. The positive control, co-amoxiclav, exhibited a zone of inhibition of 31 ± 0.0 mm while any zone of inhibition was not observed for the negative control, 2% DMSO. Therefore, further investigations were conducted using the ethyl acetate extract. In addition, the MIC value of 31.25 *μ*g/mL reflected the high antibacterial potency of this ethyl acetate extract which is quite comparable with that of the positive control co-amoxiclav (MIC = 7.8 *μ*g/mL).

The ethyl acetate extract was incorporated into the gel base at three different concentrations, and all three concentrations were capable of inhibiting the growth of *S. aureus* and *P. acne*, as visualized by distinct zones of inhibitions in agar plates.

### 3.2. Qualitative Screening for the Phytochemical Constituents in the Ethyl Acetate Extract Prepared from Seeds of *N. sativa*

The qualitative phytochemical analysis revealed the presence of alkaloids, phenolics, and flavonoids in the above extract.

### 3.3. Antibacterial Activity in the Gel Formulations

**Figure 1:**
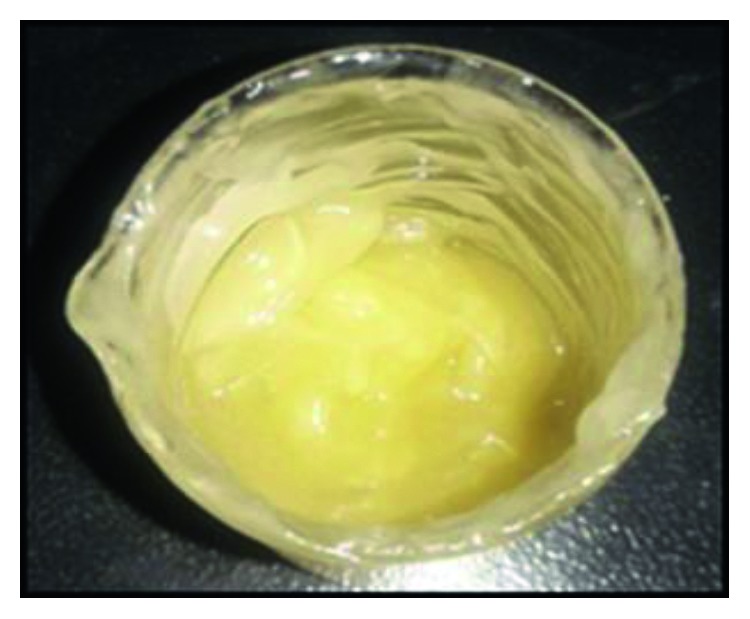
Appearance of the gel formulation F3.

As indicated in Table 2, the formulation F3 with 15% of the seed extract (Figure 1) displayed the highest activity against both bacterial species. The negative controls, i.e., gel base and methanol, did not exhibit any inhibition while zones of inhibition with a diameter of 8.3 ± 1.5 and 10.5 ± 0.7 were observed for the synthetic commercial antiacne gel product (comprised of sulfur, isopropylmethylphenol, stearyl glycyrrhetinate, vitamin E, and vitamin B6 as active ingredients) against *S. aureus* and *P. acnes*, respectively.

The MIC values against *S. aureus* were observed as 250, 62.5, and 62.5 *μ*g/mL for F1, F2, and F3, respectively. Interestingly, the MIC value of the synthetic commercial antiacne gel was observed as 125 *μ*g/mL, indicating a potent antibacterial activity in formulations F2 and F3 against *S. aureus* in comparison with this synthetic product. Although the same MIC values were observed for F2 and F3 against *S. aureus*, the diameter of the zone of inhibition was slightly higher in F3 in comparison with F2 as observed in the agar well diffusion assay.

### 3.4. Stability of the Physical Parameters and the Antibacterial Activity

All three gel formulations demonstrated a good stability, without any change in the status of the initial physical parameters over an experimental period of 30 days (Table 3).

Interestingly, the findings proved that the antimicrobial property of these gel formulations against *S. aureus* has retained during the storage period as evidenced by the presence of zones of inhibition of 10.5 ± 0.7, 11.5 ± 0.7, and 12.0 ± 0.0 mm in diameter for formulations F1, F2, and F3, respectively.

### 3.5. Acute Local Irritation Test with the Optimized Gel Formulation

Out of the 50 participants, only 7 (14%) have developed some signs of hypersensitivity while the majority of the participants (86%) were unaffected by the application of the herbal gel formulation.

## 4. Discussion

The microbial flora isolated from acne patients include *P. acnes*, *S. epidermidis*, *S. aureus*, *Klebsiella pneumonia*, and *Streptococcus* whose pathogenic mechanisms and the genes associated with virulence factors are believed to play a significant role in the development of acne [19]. Although antibiotics have been included in the therapeutic regimes of acne, the number of reports on antimicrobial resistance by acne-causing bacterial species is escalating over the recent years. For example, in Europe and in the United States, antimicrobial-resistant *P. acnes* strains have been isolated frequently while a study conducted in Japan revealed a relationship between the use of antimicrobial agents and the emergence of antimicrobial resistance against *P. acnes*. Furthermore, the analysis of correlation between the antimicrobial resistance of *P. acnes* and *S. epidermidis* had revealed that more than 80% of the patients who carried clindamycin-resistant *P. acnes* also carried clindamycin-resistant *S. epidermidis* [20]. Apart from that, the adverse effects associated with benzoyl peroxide, retinoids, isotretinoids, azelaic acid, and salicylic acid and other widely used antiacne agents could not be neglected [21]. This necessitates the development of novel therapeutic agents with high efficacy and low side effect profiles. In this approach, a number of plant extracts and phytochemicals thereof have been reported with antibacterial activity against acne-causing bacterial species. For example, the extracts of *Punica granatum*, *Morus alba*, and *Angelica anomala* have exhibited MIC in the range of 4–50 *μ*g/mL against *P. acnes*, along with a potent antibacterial activity against *S. epidermidis*. Similarly, MICs of 0.005–0.6 *μ*L/mL have been reported in essential oils of *Citrus obovoides*, *Citrus natsudaidai*, *Cryptomeria japonica*, and *Cymbopogon nardus*, while phytochemicals such as pulsaquinone, hydropulsaquinone, rhodomyrtone, and rhinacanthin-C were found to possess MIC in the range of 0.5–12.5 *μ*g/mL against *P. acnes* [22]. These observations suggest that medicinal plants could be potential sources of novel pharmaceuticals for treating acne. Hence, the present investigation was undertaken to develop topical formulations which are effective against acne-causing bacterial species using seed extracts of *N. sativa*, a plant that has been reputed in Sri Lankan folklore medicine as a remedy for acne, eruptions of the skin, and related skin diseases [15].

Despite the availability of several reports on antibacterial effect of the crude extracts prepared from *N. sativa* against acne-causing bacterial species [23], the effect of topical gel formulations comprising *N. sativa* on those microorganisms is yet to be documented. For instance, the antibacterial potential of methanolic extract of seeds of *N. sativa* was evaluated against *P. acnes* in a recent study; however, the gel formulation prepared with the above extract was not subjected to the relevant antimicrobial assays [24]. Thus, the present study provides new insight into the possible antiacne effect of topical gel formulations incorporated with the extracts of *N. sativa*. Moreover, it demonstrates several advanced features that were lack in the gel formulation reported by Bhalani and Shah [24]. This includes the incorporation of cetyl alcohol, an emollient, and moisturizer to reduce the inherent irritating property in *N. sativa*, as well as fuller's earth for the absorption of excess sebum present in the facial skin. Noteworthy, our formulations are free of paraben, a common chemical constituent employed in cosmetic industry, but has been frequently subjected to discussions on its carcinogenic potential. Moreover, phenoxyethanol was included as the preservative along with EDTA to stabilize the formulation from rancidity and consequently to enhance the aesthetic appeal of the product as well as the water washability of the gel. As the vehicle, rosewater was used in these novel formulations and it is assumed that rosewater could assist in maintaining the pH while reducing erythema, dermatitis, and eczema, owing to its anti-inflammatory potency. Furthermore, the pH value of the formulations prepared in this study lies in the range of 5–6 and it accords with the optimal pH of the skin which is accepted as 5.5.

Interestingly, all three formulations developed in this study displayed a good stability with respect to a number of physical parameters while retaining its antibacterial potential, although this feature has not been tested in the previous study by Bhalani and Shah [24]. Out of the three formulations, the antibacterial activity was conspicuous in the formulation prepared with 15% of the extract, and its potency against *S. aureus* was superior to the commercial synthetic product employed as the positive control. On the other hand, the comparison of MIC values of the gel formulations and the positive control against *P. acnes* could have improved the outcome of this study; however, the limited facilities in the current laboratory setup and the unavailability of validated and reliable experimental protocols have hindered the determination of MIC for *P. acnes*. This was the major limitation in the present study. Nevertheless, the present investigation demonstrated the significance of these novel gel formulations prepared with seed extracts of *N. sativa* as curative and palliative products for acne vulgaris. This is supported by the results of acute local irritation test in which the occurrence of hypersensitivity reactions upon the application of the optimized gel formulation was found to be minimal as only 14% of tested volunteers had developed some signs of allergic symptoms. However, further investigations are warranted with a larger number of healthy human volunteers to determine any adverse effects that could be associated with the application of these formulations on human skin and thereby to determine the suitability to develop as commercial products. Since genotoxic and cytotoxic effects have been observed at or above 2 and 5 *μ*g/mL of the extracts prepared from *N. sativa*, respectively [25], detailed cytotoxicity assays are required to confirm that the concentrations of the extract incorporated into the topical formulations do not impose any cytotoxic or genotoxic effects. Yet, the preliminary findings of this study make significant contribution to the field of alternative antiacne remedies, particularly for the development of herbal formulations with several advanced features that have not been reported in the literature before. Moreover, the strong antimicrobial activities observed against some acne-causing microorganisms would provide solid scientific evidence for the usage of *N. sativa* in antiacne formulations in indigenous medicine in Sri Lanka.

## 5. Conclusion

The topical gel formulations from seeds of *N. sativa* were successfully formulated and evaluated for different parameters including the antibacterial activity. The results indicate that the prepared formulations have strong antibacterial potency against acne-causing bacteria and the activity was prominent in the formulation comprising 15% of the extract. Interestingly, the *in vitro* antibacterial potency of this formulation surpassed that of the synthetic commercial antiacne formulation while the number of individuals who developed hypersensitivity reactions upon the application of the optimized formulation was found to be insignificant. Hence, the present investigation revealed the possibility of developing commercial products with *N. sativa* for the management of acne vulgaris while rationalizing its utility as an antiacne remedy in Sri Lankan traditional medicine.

## Figures and Tables

**Table 1:** Ingredients of gel formulations F1, F2, and F3 (in grams per 100 grams of the formulation).

Ingredient	Weight of the ingredients in the formulations (in grams)		
	F1	F2	F3
Carbopol 940	1.10	1.10	1.10
Phenoxyethanol	1.00	1.00	1.00
Glycerin	3.00	3.00	3.00
Polyethylene glycol (PEG)	0.05	0.05	0.05
Triethanolamine	Quantity sufficient		
Fuller's earth	0.10	0.10	0.10
Cetyl alcohol	0.01	0.01	0.01
Ethylenediaminetetraacetic acid (EDTA)	0.10	0.10	0.10
Rosewater	Quantity sufficient		
*N. sativa* extract	5.00	10.00	15.00

**Table 2:** Antibacterial activity in three topical gel formulations and positive and negative controls against *S*. *aureus* and *P. acnes*.

Microorganism	Antibacterial activity in terms of diameter of the zone of inhibition (mm)				
	F1	F2	F3	Positive control	Negative control
*S. aureus*	8.0 ± 0.0	9.3 ± 0.5	10.6 ± 0.5	8.3 ± 1.5	0.0 ± 0.0
*P. acnes*	8.0 ± 0.0	8.0 ± 0.0	9.0 ± 0.0	10.5 ± 0.7	0.0 ± 0.0

**Table 3:** Comparison of the physical parameters of the topical gel formulations at day 1 and day 30.

Formulation no.	Number of days after formulation	Color	Odor	Homogeneity	Water washability	Consistency	pH
F1	Initial	Pale yellow	Pleasant	Homogeneous	Washable	Semisolid	5-6
	30	Pale yellow	Pleasant	Homogeneous	Washable	Semisolid	5-6

F2	Initial	Yellow	Pleasant	Homogeneous	Washable	Semisolid	5-6
	30	Yellow	Pleasant	Homogeneous	Washable	Semisolid	5-6

F3	Initial	Dark yellow	Pleasant	Homogeneous	Washable	Semisolid	5
	30	Dark yellow	Pleasant	Homogeneous	Washable	Semisolid	5

## Data Availability

The data used to support the findings of this study are included within the article.
